# Colorectal cancer liver metastases: mechanism and therapy

**DOI:** 10.3389/fimmu.2026.1861130

**Published:** 2026-07-10

**Authors:** Shan Liu, Liuxian Ban, Wei Zhang, Yutian Sun, Ke Wang, Yuxin Man, Yinjie Zhang

**Affiliations:** 1 Department of Radiation Oncology, Sichuan Clinical Research Center for Cancer, Sichuan Cancer Hospital & Institute, Sichuan Cancer Center, University of Electronic Science and Technology of China, Chengdu, China; 2 Department of Clinical Oncology, Li Ka Shing Faculty of Medicine, The University of Hong Kong, Hong Kong, China; 3 Clinical Oncology Center, Shenzhen Key Laboratory for Cancer Metastasis and Personalized Therapy, The University of Hong Kong-Shenzhen Hospital, Shenzhen, China; 4 Department of Medical Oncology, Sichuan Clinical Research Center for Cancer, Sichuan Cancer Hospital & Institute, Sichuan Cancer Center, University of Electronic Science and Technology of China, Chengdu, China

**Keywords:** colorectal cancer liver metastases, hepatic immune microenvironment, immunosuppressive niche, immunotherapy resistance, MSS/pMMR

## Abstract

Colorectal cancer (CRC) is a leading cause of cancer mortality worldwide, with liver metastasis representing the principal cause of death in CRC patients. Current primary treatment options for colorectal cancer liver metastases (CRLM) encompass chemotherapy, radiotherapy, targeted therapy, and immunotherapy. However, the survival rate for CRLM patients remains below 40%. The unique immune−tolerant microenvironment of the liver has recently been recognized as a central driver of metastatic colonization, immune evasion, and therapeutic resistance. This review examines the development of CRLM, with particular emphasis on recent therapeutic advances. It also explores promising research and emerging treatment strategies. A deeper understanding of CRLM pathogenesis and therapeutic progress will enable clinicians to optimize treatment approaches and improve patient outcomes.

## 1 Introduction

Colorectal cancer (CRC) stands among the most prevalent and lethal malignancies on a global scale. According to the 2024 global cancer statistics released by the WHO, CRC ranks as the third most commonly diagnosed cancer worldwide, surpassed only by breast and lung cancers. The disease burden is substantial. Each year, it accounts for more than 1.9 million new diagnoses and claims approximately 900,000 lives (1). The high mortality rate of CRC is mainly related to its distant metastasis, in which the liver is a common site. It is reported that about 50% patients undergo colorectal cancer liver metastases (CRLM) when the disease progresses. CRLM is one of the major causes of death in CRC patients, accounting for approximately 70% of the fatal cases (2). Besides, liver-specific recurrence exhibits a high rate, affecting 50-75% of patients who previously had their liver metastases resected (3).

Anatomically, the liver receives a dual blood supply from the portal vein and hepatic artery, with slow blood flow and highly permeable sinusoidal endothelium, which provides favorable conditions for the arrest and extravasation of circulating tumor cells (4). Immunologically, the liver is a naturally immune−privileged organ, enriched in Kupffer cells (KCs), regulatory T cells (Tregs), and myeloid−derived suppressor cells (MDSCs), and constitutively expresses immune checkpoint molecules such as PD−L1 (5). This tolerogenic environment is primarily evolved to prevent excessive inflammatory responses against food antigens and gut commensals (6). Unfortunately, it is readily hijacked by colorectal cancer cells to construct an immunosuppressive “soil” that favors metastatic colonization (4).

**Figure 1:**
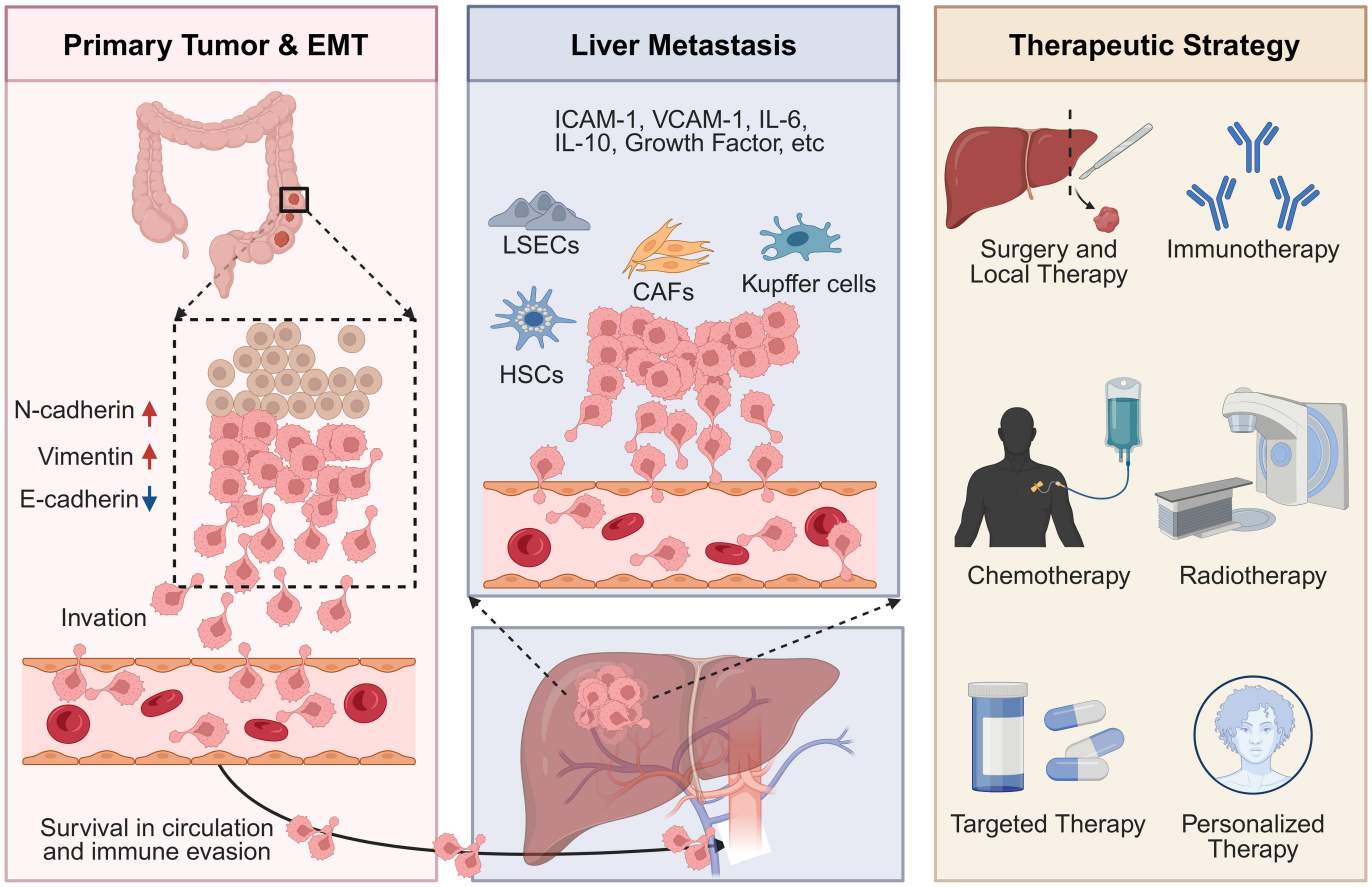
Overview of the pathogenesis and therapeutic management of CRLM. Shown is a diagrammatic representation of the intricate process by which colorectal cancer tumor cells break away from the primary tumor, traverse through the circulatory system, and ultimately establish metastatic colonies in the liver. This illustration also encompasses an overview of the current therapeutic strategies employed in the management of colorectal cancer liver metastasis. LSECs, Liver sinusoidal endothelial cells; CAFs, Cancer-associated fibroblasts; HSCs, Hepatic stellate cells. Image created in https://BioRender.com.

In this review, we will elaborate on the cellular and molecular mechanisms underlying CRLM and systematically dissect the multifaceted factors by which this immunosuppressive niche drives resistance to ICIs in the vast majority of microsatellite−stable/mismatch repair−proficient (MSS/pMMR) patients (**Figure 1**). Concurrently, we propose a dynamic three−stage integrated model and incorporate metabolic reprogramming into the discussion of the immunosuppressive network. These insights may contribute to a deeper understanding of the hepatic immune microenvironment and provide a reference for the future design of combination therapeutic strategies.

## 2 Potential molecular and cellular mechanisms underlying crlm

CRLM develops through a multistep biological process involving primary tumor invasion, survival in circulation, immune evasion, and eventual colonization of the liver. As the primary tumor progresses, cancer cells undergo an epithelial-mesenchymal transition (EMT), losing epithelial adhesion and acquiring mesenchymal characteristics. This transition is marked by the E-cadherin downregulation and N-cadherin and Vimentin upregulation. At the molecular level, these alterations enhance the migratory and invasive capacity of the cancer cells (7). Cancer cells then enter the hepatic portal circulation by the mesenteric venous system. In the circulation, most cells perish due to shear stress and immune surveillance. Only a minority adhere to hepatic sinusoidal endothelial cells and activate adhesion molecules like intercellular adhesion molecule-1 (ICAM-1) and vascular cell adhesion molecule-1 (VCAM-1) to establish initial colonization (7).

Concurrently, the hepatic microenvironment undergoes remodeling to establish a pre-metastatic niche (PMN) centered on an immune-suppressive network. The PMN refers to a microenvironment pre-established by the primary tumor in distant organs through the secretion of factors and exosomes, which favors the colonization and growth of tumor cells (8). In the CRLM, the liver serves as a “hotspot” organ for PMN formation due to its unique anatomical features (dual blood supply, highly permeable endothelium) and immune tolerance characteristics (9). The establishment of the hepatic PMN represents a critical step in the pathogenesis of CRLM. This process preconditions the liver, transforming it into fertile “soil” conducive to metastasis. The “seed” of tumor cells arriving at the liver and the “soil” of the liver are mutually interdependent, jointly driving metastatic colonization. The following sections will provide a detailed analysis of the core components constituting the PMN, which will then be integrated into a dynamic three-stage model in Section 2.5.

### 2.1 Cellular architects of the immunosuppressive niche

**Figure 2:**
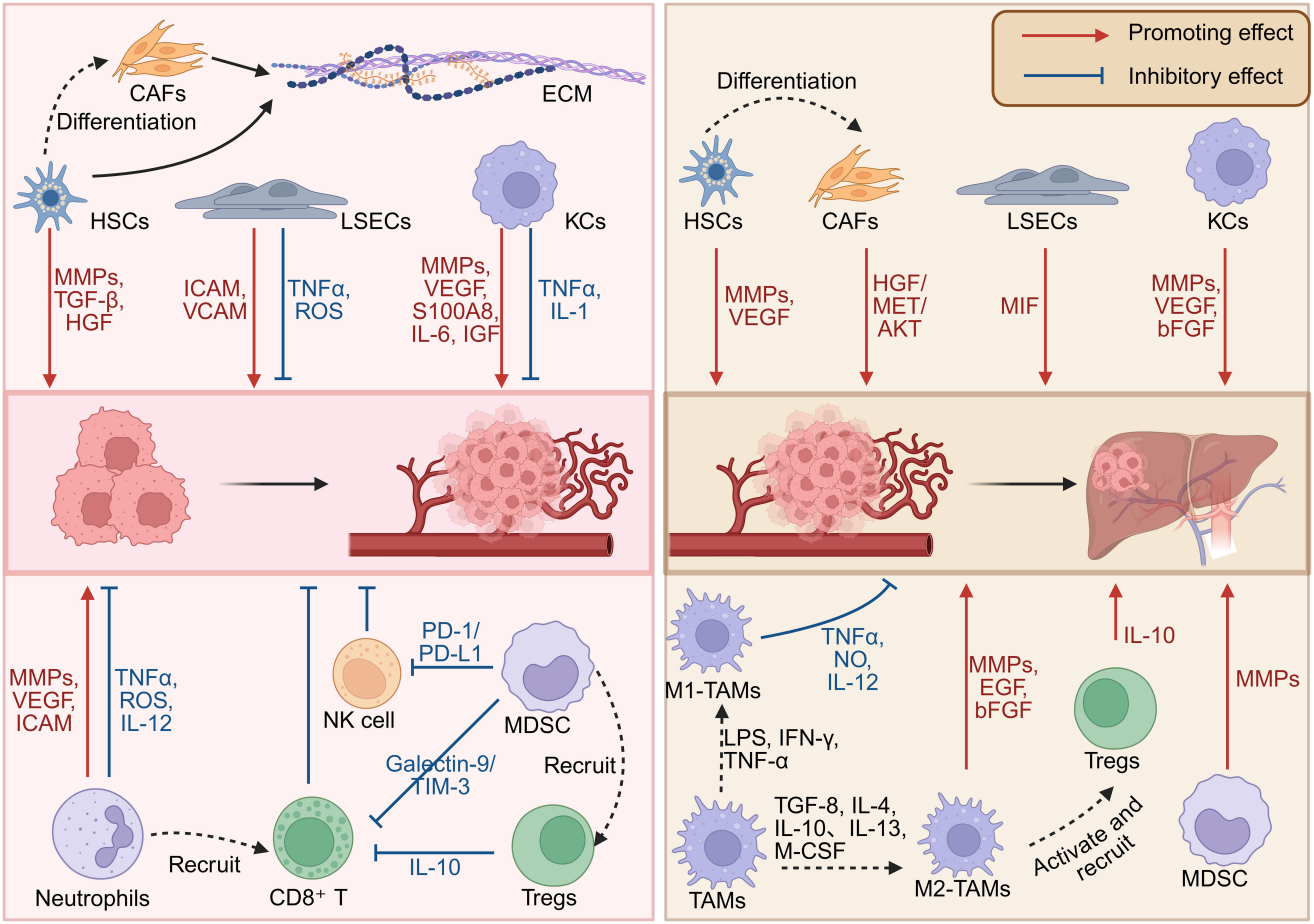
The role of different cells of the hepatic microenvironment in colorectal cancer liver metastases. Shown is a diagrammatic representation of how various cells secrete cytokines to promote or inhibit the processes of angiogenesis and tumor colonization. HSCs, Hepatic stellate cells; LSECs, Liver sinusoidal endothelial cells; CAFs, Cancer-associated fibroblasts; KCs, Kupffer cells; ECM, Extracellular matrix; MDSC, Myeloid-derived suppressor cells; MMPs, Matrix metalloproteinases; HGF, Hepatocyte growth factor; ICAM, Intercellular adhesion molecule; VCAM, Vascular cell adhesion molecule; VEGF, Vascular endothelial growth factor; MIF, Macrophage migration inhibitory factor; bFGF, basic fibroblast growth factor. Image created in https://BioRender.com.

In CRLM, the immunosuppressive niche is a core subset of the PMN. It refers to a local microenvironment co-constructed by tumor cells, stromal cells, and immune cells. And it characterized by effective suppression of anti-tumor immune responses and enhancement of pro-tumor inflammation, angiogenesis, and matrix remodeling. Consequently, this niche enables tumor cells that have reached the liver to evade immune surveillance, survive, proliferate, and ultimately form metastatic colonies (**Figure 2**).

#### 2.1.1 Matrix remodeling: hepatic stellate cells, cancer-associated fibroblasts, and extracellular matrix stiffening

Hepatic stellate cells (HSCs), a resident type of mesenchymal stromal cell, reside in the perisinusoidal space of Disse and constitute approximately 10% of all liver cells. Following activation by tumor-derived exosomes (miR-181a-5p), TGF-β, and IL-6, HSCs undergo transdifferentiation into cancer-associated fibroblasts (CAFs) (10). Subsequently, activated HSCs secrete periostin via the αvβ3 Integrin-Akt/PKB pathway, which promotes the survival of tumor cells and endothelial cells in the liver. Furthermore, activated HSCs secrete MMP-8 and MMP-9, which activate growth factors and stimulate tumor growth and angiogenesis in CRLM (11).

CAFs further exacerbate immunosuppression. TGF-β, IL-6, and other signals released by CAFs induce interstitial fibrosis and increased matrix stiffness, remodeling the extracellular matrix (ECM) (12). On one hand, excessive ECM deposition and enhanced collagen crosslinking lead to elevated tissue stiffness, which activates the YAP/TAZ pathway via the integrin/focal adhesion kinase (FAK) signaling axis. This process upregulates PD-L1 expression, thereby enhancing immune evasion in tumors (13, 14). Strategies targeting the integrin-FAK-YAP axis have demonstrated potential for reversing resistance to immune checkpoint inhibitors (ICIs) in preclinical studies. They can downregulate YAP activity, thereby facilitating cytotoxic T cell infiltration and potentiating antitumor immunity (15). On the other hand, matrix stiffness significantly inhibits T cell activation, cytokine production, and proliferation through YAP signaling (16). Meanwhile, the dense collagen network secreted by CAFs forms a physical barrier that restricts the movement of CD8^+^ T cells into tumor nests (17). Therefore, within the microenvironment of CRLM, YAP/TAZ-mediated immune evasion and the physical barrier constructed by CAFs interact and synergize, collectively shaping an immunosuppressive tumor microenvironment.

Despite ample preclinical evidence, clinical trials targeting CAFs, such as those employing anti-FAP antibodies (NCT04826003) or FAK inhibitors (NCT01138033), have largely failed to achieve expected efficacy, which likely reflects the functional heterogeneity of CAFs (18). Single-cell transcriptomic studies have revealed that CAFs in the CRLM liver microenvironment can be divided into functionally opposing subsets, including myofibroblastic CAFs (myCAFs) and inflammatory CAFs (iCAFs) (19). myCAFs secrete hyaluronic acid that promotes tumor growth, yet the expression of type I collagen may suppress tumor expansion through mechanical constraint. In contrast, iCAFs drive tumor progression via the HGF-MET axis (20). Therefore, distinct CAF subsets may exert both pro-tumor and anti-tumor effects in CRLM. Future therapeutic strategies must deeply dissect the functional spectrum of CAF subsets and precisely target specific pro-metastatic subsets rather than non-selectively eliminating all CAFs.

#### 2.1.2 Endothelial and innate immune barrier: liver sinusoidal endothelial cells and Kupffer cells

Liver sinusoidal endothelial cells (LSECs) play a complex dual role in CRLM. After CRC cells evade mechanical stress and immune surveillance to enter the space of Disse, LSECs can directly eliminate them through endocytosis and initiate antigen presentation. LSECs also secrete inflammatory factors, including IFNγ, NO, ROS, and TNFα, which induce CRC apoptosis via the Fas/FasL pathway (21). On the other hand, LSECs can upregulate the expression of adhesion molecules, including VCAM-1, ICAM-1, E-selectin, integrins, and cadherins. These molecules facilitate tumor cell retention and transport by strengthening their adhesion to the LSEC surface (22).

Moreover, bidirectional signaling between tumor cells and LSECs fosters a pro-metastatic microenvironment. Tumor-derived ligands, including CD44, sLewA, and sLewX, bind to E-selectin on LSECs to form adhesion complexes that facilitate CRC liver colonization. This interaction further amplifies LSEC activation by stimulating high mobility group box 1 (HMGB1) secretion, thereby establishing a positive feedback loop (23). At the immune level, activated LSECs upregulate PD-L1, secrete IL-10, and express adhesion molecules, thereby directly inducing the differentiation of Tregs and suppressing effector T cell function (9, 24). Therefore, upon encountering tumors, LSECs undergo a functional shift from anti-tumor immune surveillance to the induction of immune resistance.

As the resident macrophages of the liver, KCs also assume a dual role in CRLM. During early metastasis, KCs exert anti-tumor effects via phagocytosis and by releasing cytokines including TNF-α and IL-1α, alongside oxygen metabolites and proteases. This activity activates neutrophils and NK cells, thereby inhibiting CRLM progression (25). In a state of tolerance, however, KCs induce the expression of adhesion molecules on LSECs and secrete factors like IL-6, MMPs, and VEGF, which promote tumor cell retention, invasion, and proliferation (26). Furthermore, through antigen presentation, KCs can activate Tregs and upregulate PD-L1 expression, consequently suppressing anti-tumor immunity (27).

Notably, KCs are the primary phagocytes responsible for the uptake of tumor−derived exosomes in the liver (28). Tumor−derived exosomes reprogram KCs toward an M2−like phenotype through multiple mechanisms. For instance, exosomal miR−21−5p and miR−155−5p have been shown to induce M2 polarization of macrophages (29). Additionally, exosomal miR−934 drives M2 macrophage polarization by downregulating PTEN expression and activating the PI3K/AKT signaling pathway, thereby promoting liver metastasis of colorectal cancer (30). Recent studies have further demonstrated that hypoxic CRC cells secrete exosomes carrying the mutant KRAS protein. Upon uptake by KCs, these exosomes induce M2−like polarization through hyperactivation of the AKT signaling pathway, which in turn accelerates liver metastasis formation (31). Moreover, M2−polarized KCs recruit CXCR2^+^ MDSCs by secreting chemokines such as CXCL5, thereby amplifying local immunosuppression (26). In preclinical models, AKT inhibitors have shown the potential to block KC M2 polarization and suppress liver metastasis (31). However, KCs also exert anti−tumor effects during the early stages of metastasis, and complete KC depletion may exacerbate inflammation−driven metastatic progression. Therefore, future therapeutic strategies should focus on the selective reprogramming of M2−like KCs or the inhibition of specific pro−metastatic molecules, aiming to preserve the anti−tumor potential while attenuating the immunosuppressive functions.

#### 2.1.3 Myeloid and adaptive immune suppression: tumor-associated macrophages, regulatory T Cells, and myeloid-derived suppressor cells

Within the immunosuppressive niche of CRLM, tumor-associated macrophages (TAMs), Tregs, and MDSCs form an interdependent immunosuppressive network. These cell populations are tightly coupled through cytokines, chemokines, and metabolic products, collectively maintaining the immune−tolerant state of the liver microenvironment.

TAMs are pivotal in fostering an immunosuppressive microenvironment. TAMs encompass pro-inflammatory M1 macrophages and anti-inflammatory M2 macrophages. In the context of liver metastasis, TAMs are recruited to the TME via CCL2 and subsequently polarized to the M2 phenotype under the Wnt/β-catenin signaling pathway and various cytokines, including TGF-β, IL-4, IL-10, IL-13, and macrophage colony-stimulating factor (M-CSF) (32). M2 macrophages exert immunosuppressive functions through two pathways. On the one hand, M2 macrophages can release prostaglandin E2 (PGE2), IL-10, and indoleamine 2,3-dioxygenase (IDO) to activate Tregs. Besides, M2 macrophages can recruit Tregs through the secretion of chemokines CCL17, CCL18, and CCL22 (32). On the other hand, M2 macrophages promote extracellular matrix remodeling by secreting MMPs, components of the plasminogen activation system, and kallikrein−related peptidases, and directly promote tumor growth and angiogenesis by secreting pro−tumorigenic growth factors such as VEGF, epidermal growth factor (EGF), and basic fibroblast growth factor (bFGF) (33).

However, the binary M1/M2 classification framework is being refined by more granular lineage studies. Single−cell RNA−sequencing has revealed that macrophages in CRLM actually exhibit a continuous functional spectrum rather than a dichotomous polarization. Of particular interest is the metastasis−associated macrophage (MAM) subset, which co−expresses the pro−inflammatory marker TNF−α together with the immunosuppressive molecules IL−10 and PD−L1, representing a mixed functional state (34). The existence of such a functional continuum may explain why global depletion strategies targeting M2−like macrophages have repeatedly encountered setbacks in clinical practice. This finding suggests that therapeutic goals should not be a simple binary switch but rather shift toward more nuanced regulation across a functional gradient.

In addition to TAMs, Tregs also reinforce the immunosuppressive network through their unique mechanisms. High Treg infiltration is associated with the clinical outcomes of CRLM. A retrospective clinical study of 188 patients with CRLM demonstrated that a higher clinical risk score correlated with greater intratumoral Treg infiltration. High Treg infiltration was identified as an independent predictor of shorter overall survival in CRLM (35). Another study demonstrated that during the progression of CRLM, there is an increased accumulation of TNFR2-dependent Tregs. In TNFR2-deficient mice, the numbers of CD4+FoxP3+ Tregs and CD11b+GR-1+ MDSCs were significantly reduced, accompanied by a marked decrease in liver metastases (36). Further exploration of the mechanism revealed that Tregs can release IL-10, which induces PD-L1 expression on monocytes, thereby suppressing the anti-tumor effect of T cells (37). Notably, the liver is physiologically enriched with Tregs. Tumor cells further expand this endogenous Treg pool via the CCL22/MDSC intermediate, amplifying the intrinsic immune resistance mechanism to a pathological level and exacerbating local immunosuppression.

Meanwhile, the accumulation of MDSCs is also a hallmark of CRLM. Studies have shown that in CRC, tumor cells or macrophages can promote the recruitment of MDSCs through cytokines such as IL-10 and TGF-β, as well as via the CCL15-CCR1 axis and the CCRK-CXCL1 axis (38). Furthermore, MDSCs induced by the S1PR1-STAT3 pathway in CRC cells suppress T cell proliferation, which facilitates the formation of a pre-metastatic microenvironment within the liver and promotes liver metastasis (39). These MDSCs can express MMP9, MMP2, and PD-L1, thereby promoting tumor cell metastasis and immune suppression. The galectin-9 expressed by MDSCs binds to the inhibitory receptor TIM-3, inducing apoptosis of effector T cells and attenuating anti-tumor effect (40).

In summary, M2−polarized TAMs, Tregs, and MDSCs do not function as isolated immune populations. Instead, they cooperate with each other to collectively constitute an immunosuppressive ecosystem. Therefore, future therapeutic strategies targeting this system should focus on selective reprogramming rather than simple depletion. Examples include targeting the functional polarization of M2−like TAMs, blocking MDSC recruitment and metabolic activity, or interfering with NET formation and signaling. These approaches aim to reverse immunosuppression while preserving the intrinsic immune surveillance capacity of the liver.

### 2.2 Gene mutations and niche remodeling: from driver mutations to immune evasion

Genetic alterations in CRLM not only confer proliferative or invasive advantages to tumor cells but also fundamentally reprogram tumor–immune interactions, converting hepatic immune surveillance into an immune-privileged metastatic niche.

*APC* mutations drive immune exclusion by activating the Wnt/β-catenin signaling pathway. Mutation of the tumor suppressor gene *APC* is an early event in CRC. Studies have shown that the Wnt/β-catenin signaling pathway is activated in APC-mutant CRC. This pathway functions by reducing IL-1β expression, which in turn hinders the maturation of dendritic cells (DCs) in the tumor microenvironment (TME), and ultimately promotes tumorigenesis and progression (41). In the liver, the functional loss of DCs prevents cross−presentation of tumor antigens to CD8^+^ T cells, creating an immune desert before overt metastatic colonization. Furthermore, this pathway elevates nuclear β-catenin levels, enabling it to complex with T-cell factor/lymphoid enhancer factor (TCF/LEF) and regulate target gene expression, including Axin2, SOX4, TCF7, c-Myc, and MMP7 (42). This regulatory mechanism promotes both EMT and the self-renewal capacity of cancer stem cells (CSCs).12 A recent study demonstrated that transmembrane 4 L six family member 1 (TM4SF1) modulates Wnt/β-catenin signaling to regulate Sox2 expression via c-Myc in CRC, thereby promoting cell migration and metastasis (43).

*KRAS* mutations promote the formation of an immunosuppressive microenvironment by reprogramming the liver stroma. The study found that patients with multiple liver metastases (34% vs. 18%, P = 0.02) and those who developed liver metastases within one year (36% vs. 14%, P = 0.005) exhibited a higher *KRAS* mutation rate (44). The *RAS* gene family comprises *KRAS*, *NRAS*, and *HRAS*, with *KRAS* being the most frequently mutated subtype in CRC. *KRAS* mutations occur in approximately 30-50% of CRC cases, whereas *NRAS* mutations are found in 3-5% of patients (45). The most common mutations are located at codons 12, 13, and 61. These mutations result in the constitutive activation of the RAS protein, which remains GTP-bound independently of upstream EGFR signaling. Consequently, the MAPK and PI3K-AKT-mTOR pathways are persistently activated, enhancing cell proliferation and invasion (46). *RAS* mutations also activate the transcription factor CP2 (TFCP2), which drives the differentiation of CAFs into lipid-rich cells. This differentiation facilitates abundant VEGFA production, promoting tumor angiogenesis to supply the nutrients and pathways required for rapid tumor growth and metastasis (45, 47). Notably, this angiogenic support is inseparable from immunosuppression. VEGFA not only promotes angiogenesis but also directly inhibits dendritic cell maturation and facilitates regulatory T cell recruitment via VEGFR2 signaling (48).

Multiple genetic mutations converge to disrupt immune recognition in CRLM. Inactivation of *TP53* promotes genomic instability and confers drug resistance. Tumors harboring co−mutations in *KRAS* and *TP53* demonstrate enhanced hepatic colonization and immune evasion (49). *SMAD4* mutation, which disrupts the TGF-β signaling pathway, serves as an independent poor-prognosis predictor in CRLM (50). Furthermore, *ERBB2* (*HER2*) amplification, found in approximately 3-5% of *RAS/BRAF* wild-type cases, represents an emerging therapeutic target (51).

Genetic mutations in CRLM should not be viewed merely as tumor−intrinsic drivers; they can also be understood as systemic reprogramming instructions that pre−emptively disrupt hepatic immune surveillance. A comprehensive analysis of the genetic mechanisms in CRLM is therefore essential for precisely identifying potential targets and could inform the development of novel treatments.

### 2.3 Tumor-derived exosomes

Exosomes are lipid bilayer vesicles 30–100 nm in diameter containing proteins, miRNAs, lncRNAs, and mRNAs. They function as long−range communication mediators between the primary CRC and the liver, pre−shaping the hepatic immune microenvironment before tumor cell arrival (**Table 1**).

**Table 1:** The substances of exosomes and their roles in CRLM.

Substances	Roles	Reference
ANGPTL1	Suppress the JAK2-STAT3 signaling pathway and decrease MMP9 production in Kupffer cells	(55)
WNT	Induce CRC stem cell activity	(97)
TGF-β1	Remodel the liver pre-metastatic niche and promote tumor liver metastasis	(98)
IL-6, Akt, and TNF-α	Induce EMT in tumor cells	(98)
miR-25-3p	Increase vascular permeability and promote angiogenesis	(52)
miR-21-5p	Promote CRLM via the miR-21-Toll-like receptor 7-IL-6 axis	(56)
miR-135a-5p	Inhibit the activation of CD4^+^ T cells and enhance cellular adhesion	(56)
miR-210	Promote EMT	(99)
miR-19a, miR-19b, miR-4437, miR-23a, miR-320a and miR-92a	Related to the CRLM	(100)
miR-92a-3p	Inhibit mitochondrial apoptosis by activating the Wnt/β-catenin pathway, and inhibit FBXW7 and MOAP1, thereby enhancing stemness, EMT, and metastasis	(101)
miR-142-3p	Inhibit Numb expression and increase the number of CSCs	(102)
miR-1246/92b-3p/27a-3p and CXCL16	Promote CRC migration	(57)
miR-21-5p and miR-155-5p	Promote CRC migration	(29)
miR-203	Promote the differentiation of monocytes into M2 macrophages, and facilitate the formation of the liver pre-metastatic niche	(103)
miR-934	Activate the PI3K/Akt signaling pathway and induce M2 macrophage polarization	(103)
miR-25-3p	Silence KLF2 and KLF4, upregulate VEGFR2, downregulates ZO-1, occludin, and claudin-5, thereby enhancing vascular permeability and inducing angiogenesis	(104)

Exosomes initiate pre−metastatic niche formation through multiple mechanisms. miR−25−3p increases vascular permeability and promotes angiogenesis by silencing KLF2/KLF4, upregulating VEGFR2, and downregulating tight−junction proteins (ZO−1, occludin, claudin−5) (52). Concomitant vascular leakage facilitates fibrinogen deposition, providing a scaffold for MDSC and platelet recruitment (53). In addition, exosomal miR−203 promotes monocyte differentiation into M2 macrophages, and exosomal miR−934 induces M2 polarization by activating the PI3K/Akt signaling pathway (30, 54). CRC−derived exosomes are taken up by LSECs, KCs and hepatocytes, reprogramming the transcriptomic landscape of these cells (28).

Exosomes play a dual role in CRLM. ANGPTL1 attenuates liver metastasis by modulating KCs secretion patterns and impeding MMP9-induced vascular leakage (55). This seemingly paradoxical phenomenon may reflect temporal dynamics: exosomes released by early-stage tumors may induce KCs polarization toward a tolerant phenotype, whereas those derived from advanced tumors predominantly carry immunosuppressive molecules. Additional exosomal molecules further reinforce the immunosuppressive network: miR-21-5p promotes CRLM via the miR-21-TLR7-IL-6 axis, and exosomes derived from Fusobacterium nucleatum-infected CRC cells carry miR−1246/92b−3p/27a−3p and CXCL16, thereby facilitating tumor migration (56, 57).

Collectively, exosomes reprogram the hepatic immune environment from immune surveillance to an immunosuppressive state before tumor cell arrival, thereby promoting the establishment of CRLM.

### 2.4 Metabolic reprogramming and immune tolerance

As the metabolic hub of the body, the liver possesses a unique metabolic landscape that is hijacked by CRC cells upon invasion, leading to the construction of a profoundly immunosuppressive niche through nutrient competition and accumulation of metabolic byproducts (28).

Amino acid depletion induced by tumor cells is a key mechanism driving immune tolerance in CRLM. Under tumor stimulation, the liver upregulates the expression of IDO1, ARG1, and GCH1, which deplete tryptophan, arginine, and tetrahydrobiopterin (BH4), respectively (58). IDO1-mediated tryptophan depletion produces kynurenine, which binds to the aryl hydrocarbon receptor (AhR) on T cells, inducing Treg differentiation and effector T cell anergy (59). Additionally, the kynurenine-AhR axis upregulates PD-L1 expression, further enhancing immune evasion and promoting colorectal cancer liver metastasis (60). ARG1 depletes L-arginine, directly suppressing T cell proliferation, inducing downregulation of CD3ζ chain expression, impairing T-cell receptor signaling, and thereby leading to immunosuppression (61).

Lactate and acidosis generated by tumor glycolysis similarly and profoundly suppress anti-tumor immunity. Tumor cells produce large amounts of lactate through aerobic glycolysis, which inhibits anti-tumor immunity via multiple mechanisms. In NK cells, lactate induces intracellular acidification, leading to mitochondrial dysfunction and apoptosis (62). In macrophages, lactate promotes M2 polarization via activation of the AKT-ERK pathway, and the secreted CCL8 accelerates tumor progression (63, 64). Lactate also drives MDSC expansion through the GPR81/HIF-1α/PD-L1 axis (65). Furthermore, through epigenetic modifications such as histone lactylation, lactate suppresses effector T cells and NK cells while promoting the accumulation of Tregs and MDSCs (63).

Tumor cells also drive immune tolerance through remodeling of lipid metabolism. The liver serves as the central hub for lipid metabolism. In CRLM, activated HSCs not only induce lipid droplet formation in neutrophils via the IL33/PPARγ/DGATs signaling axis, thereby maintaining their pro−metastatic function, but also directly inhibit NK cell function through the HSC−PGE2−NK cell axis, promoting liver metastatic colonization (66, 67). Tumor−derived fatty acids and oxidized lipids further induce MDSC expansion and T−cell dysfunction (68). Additionally, HSC−derived lipid droplets provide an energy reservoir for tumor cells while generating immunosuppressive prostaglandins via cyclooxygenase−2 (COX−2), which in turn drives the accumulation of MDSCs and Tregs (69). The lipid metabolism checkpoints CD36 antibody PLT012 has shown synergistic effects with PD−1 blockade in preclinical models (70).

Metabolic interventions represent a highly promising therapeutic avenue for CRLM. The dual IDO1/TDO inhibitor HTI−1090 has entered early−phase clinical trials (NCT03208959). However, its monotherapy efficacy is limited, and combination with ICIs represents the primary direction. Targeting lactate metabolic pathways including inhibiting LDHA or blocking MCT transporters has been shown to reshape the immune landscape of the tumor microenvironment (71, 72). Targeting ARG1 can restore T−cell proliferation and function (73). Combining these metabolic intervention strategies with ICIs holds promise for relieving the metabolic checkpoint blockade on T cells and reversing immune tolerance in CRLM.

### 2.5 Pre−metastatic niche: an integrated model

Based on the mechanistic components discussed above, this section proposes a three−stage integrated model that dynamically links these components along a temporal axis.

Stage 1: Remote priming. The primary CRC releases exosomes (miR−181a−5p, TGF−β1, miR−25−3p) into the portal circulation. These exosomes reprogram LSECs to upregulate adhesion molecules (E−selectin, VCAM−1) and shift KCs toward a tolerant phenotype. Concurrently, genetic drivers in the primary tumor suppress systemic DCs function and reduce circulating effector T cells.

Stage 2: Local conditioning. Activated LSECs and KCs recruit circulating MDSCs and neutrophils via CCL2, CXCL1, and the SDF−1/CXCR4 axis. HSCs transdifferentiate into CAFs, depositing collagen and fibronectin. This ECM remodeling not only creates a physical scaffold but also sequesters IL−2 and IFN−γ, starving infiltrating T cells while providing survival signals for Tregs.

Stage 3: Symbiotic stabilization. Arriving tumor cells encounter an immunosuppressive, pro−angiogenic, and stiffened microenvironment. CAFs and HSCs provide HGF and periostin, M2−TAMs and MDSCs suppress residual T−cell activity, and Tregs maintain local tolerance. The tumor−stroma−immune tripartite establishes a self−reinforcing, drug−resistant ecosystem.

Metabolic reprogramming pervades all three stages: glycolysis fuels remote priming, lactate synergizes with ECM remodeling to suppress T cells during local conditioning, and lipid metabolic disorder consolidates immunosuppression in the symbiotic stabilization stage. The interweaving of metabolism and immunity renders this niche highly stable and refractory to treatment.

## 3 Therapeutic strategies of CRLM

**Figure 3:**
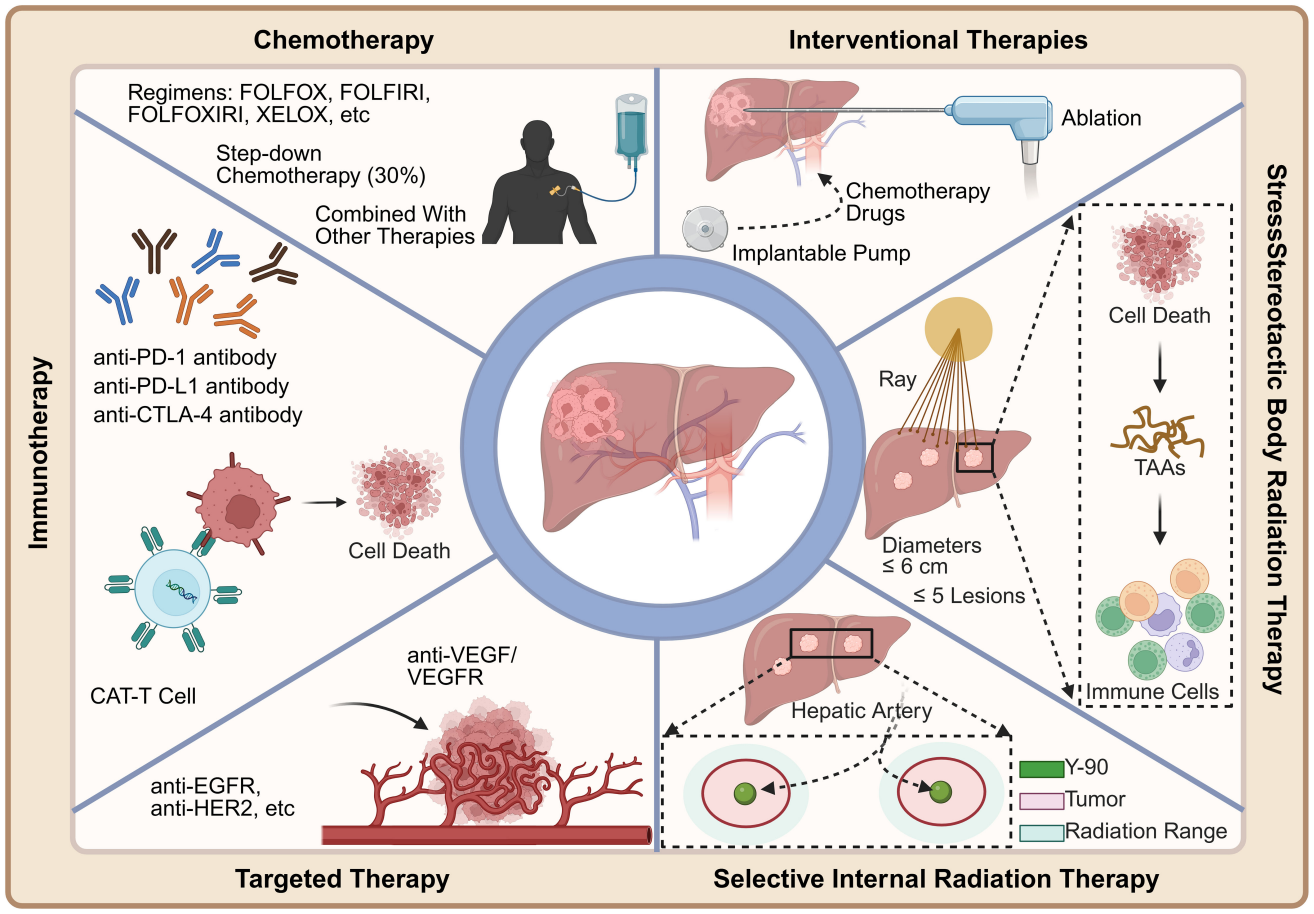
Local and systemic therapy for colorectal cancer liver metastases. CAR, Chimeric antigen receptor; CTLA-4, Cytotoxic T-lymphocyte-associated protein 4; EGFR, Epidermal growth factor receptor; PD-1, Programmed cell death protein 1; PD-L1, Programmed death-ligand 1; TAA, Tumor-associated antigen; VEGF, Vascular endothelial growth factor. Image created in https://BioRender.com.

Surgery remains the definitive treatment for CRLM. However, only 20-30% of newly diagnosed patients are eligible for resection, and the postoperative hepatic-specific recurrence rate is as high as 50-75% (74). Therefore, chemotherapy, radiotherapy, targeted therapy, and immunotherapy play crucial roles in the management of CRLM (**Figure 3**).

### 3.1 Chemotherapy

The systemic treatment of CRLM primarily relies on fluoropyrimidine-based regimens, such as FOLFOX, FOLFIRI, FOLFOXIRI, and XELOX. For CRLM that is initially unresectable, approximately 30% can be subjected to curative surgery following downstaging chemotherapy (75). Notably, the efficacy of chemotherapy combined with other therapies, such as interventional therapy and targeted therapy, in the treatment of CRLM is currently under investigation (**Table 2**).

**Table 2:** Clinical trials of treatment strategies for CRLM.

Trial ID	Phase	Population	Intervention	Result
Chemotherapy				
NCT00143403	III	Postoperative CRLM	Irinotecan + 5-FU/​FA vs. 5-FU/​FA	mDFS: 24.7 m vs. 21.6 m
NCT00006479	III	CRLM	Surgery + chemotherapy vs. Surgery	mOS: 61.3 m vs. 54.3 m; 5-year OS: 51.2% vs. 47.8%
Local treatment, with or without chemotherapy				
NCT22944367	III	CRLM	SIRT + mFOLFOX6 vs. mFOLFOX6	mPFS: 10.7 m vs. 10.2 m
NCT01839877	II	CRLM	DEBIRI + FOLFOX	9-month PFS: 53.6%; mOS: 37.4 m
NCT00440310	III	Recurrent CRLM	Litx + chemotherapy vs. Chemotherapy	mOS: 390 d vs. 405 d
NCT01098422	II	CRLM	Y-90	mPFS:7.6 m
NCT02015754	II	CRLM	DEBIRI	mOS:18.7 m
NCT01721954	III	CRLM	mFOLFOX6 + SIRT vs. mFOLFOX6	mOS: 25.9 m vs. 25.0 m; mPFS: 11.8 m vs 11.2 m
NCT00645710	I/II	CRLM	Y-90 + gemcitabine + Fudr	mPFS: 11.5 m; mOS: 73.2 m
Targeted therapy with chemotherapy				
NCT01226719	II	CRLM (KRAS-WT)	FOLFOXIRI + panitumumab	ORR: 75%
NCT00700570	II	CRLM	XELOX + bevacizumab	Conversion rate from unresectable to resectable CRLM: 42.2%
NCT22944367	III	CRLM	Cetuximab + chemotherapy vs. chemotherapy	mPFS: 22.2 m vs. 15.5 m; mOS: 81 m vs 55.4 m
NCT00778102	II	CRLM	Bevacizumab + mFOLFOX-6 vs. Bevacizumab + FOLFOXIRI	R0 rate: 23.1% vs. 48.8%
NCT00865709	II	CRLM	Sorafenib + mFOLFOX6 vs. Placebo + mFOLFOX6	mPFS: 9.1 m vs 8.7 m; mOS: 535 d vs. 552 d
NCT00803647	II	CRLM (KRAS-WT)	mFOLFOX7 + cetuximab	R0 rate: 75%

CRLM, Colorectal cancer liver metastases; MSS, Microsatellite stability; SIRT, Selective internal radiation therapy; 5-FU/FA, 5-Fluorouracil/folinic acid; DEBIRI, Drug-eluting beads irinotecan; DFS, Disease-free survival; PFS, Progression-free survival; OS, Overall survival; ORR, Objective response rate; RFS, Recurrence-free Survival.

### 3.2 Local therapies

In patients with CRLM, local therapies such as interventional procedures and radiotherapy enable the precise targeting of hepatic metastases, thereby sparing healthy tissues.

For small (< 3 cm), parenchymal lesions that do not threaten critical structures, ablation (MWA, IRE, RFA) can be an alternative to surgery, provided that incomplete ablation is avoided (76). Ablation is generally considered safer than surgery, and IRE can additionally preserve surrounding vascular, neural, and biliary structures (77). Of note, patients with RAS mutations exhibit a higher recurrence rate after ablation (78).

Hepatic artery infusion (HAI) chemotherapy exploits the fact that liver metastases are predominantly supplied by the hepatic artery, thereby achieving high−concentration drug delivery to the tumor. Compared to patients receiving systemic chemotherapy alone, those treated with systemic chemotherapy and HAI demonstrated significantly prolonged PFS (31.3 months vs. 17.2 months) and an increased 10-year overall survival rate (41.1% vs. 27.2%) (76). However, the dose of systemic chemotherapy is typically reduced by approximately 20% during HAI to mitigate toxicity, which may potentially compromise systemic control of CRC. Furthermore, HAI can lead to complications including arterial pseudoaneurysm, hepatic artery thrombosis or dissection, biliary sclerosis, hematoma, and pump infection (79). Currently, several interventional therapies combined with other treatment modalities, or novel therapeutic approaches, are under clinical investigation (**Table 2**).

Stereotactic body radiotherapy (SBRT) achieves local tumor control through hypofractionated, high−dose irradiation. The SABR-COMET clinical trial demonstrated that patients with less than 5 metastatic lesions treated with SBRT had a significantly prolonged median OS compared to those receiving standard systemic therapy (41 months vs. 28 months) (80). Radiotherapy can induce immunogenic cell death and abscopal effects. However, abscopal responses are rare, necessitating combination with ICIs to enhance systemic antitumor immunity.

Selective internal radiation therapy (SIRT) delivers internal irradiation via Y−90−loaded microspheres (81). Results from NCT01098422 showed that treatment with Y90 radioembolization yielded a median PFS of 7.6 months in CRLM patients. The results of the SIRFLOX demonstrated that the combination of SIRT to standard chemotherapy did not significantly prolong OS, but improved median PFS in the liver (20.5 vs. 12.6 months) and ORR in the liver (78.7% vs. 68.8%) (82). Y−90 therapy is generally well tolerated, and longitudinal ctDNA monitoring may predict long−term control, suggesting its value for personalized efficacy assessment.

### 3.3 Targeted therapy

In the targeted therapy of CRC, the two main pillars are inhibitors of the VEGF and EGFR, both of which are superimposed on a fluoropyrimidine-based chemotherapy backbone. Bevacizumab combined with XELOX/FOLFOX4 significantly extended median PFS (9.4 vs. 8.0 months, *P* = 0.0023) *(*83). Cetuximab plus FOLFOX improved PFS (9.2 vs. 7.4 months) and OS (20.7 vs. 17.8 months) in RAS wild−type patients (84).

However, there are some limitations to targeted therapy. Primary resistance to EGFR inhibition in right-sided primaries, RAS- or BRAF-mutant tumors and HER2-amplified disease constrains their applicability. Moreover, both EGFR and VEGF blockade are hampered by the rapid emergence of acquired resistance, mediated by secondary RAS/EGFR mutations, ligand upregulation, activation of bypass pathways such as MET or HER2, and microenvironmental adaptation (85). These limitations have catalyzed shift towards refined molecular stratification and multi-targeted combination strategies. Routine multigene panel testing now extends to incorporate ERBB2, NTRK fusions, rare ALK/ROS1 rearrangements, and emerging targets such as HER3, FGFR, and CLDN18.2 (86). Anti-HER2 strategies have shown clinically meaningful activity in ERBB2-amplified metastatic CRC. These strategies encompass the use of monoclonal antibodies, dual HER2 blockade, and antibody-drug conjugates (87). For BRAF^V600E^ mutant tumors, triplet combinations incorporating BRAF and EGFR inhibitors have improved outcomes. Inhibitors targeting KRASG12C/G12D, SHP2, and ERK inhibitors (88).

### 3.4 Immunotherapy

In CRLM patients, immunotherapy mainly includes immune checkpoint inhibitors (ICIs) and chimeric antigen receptor (CAR) T-cell therapy.

#### 3.4.1 Immune checkpoint inhibitors

ICIs primarily comprise anti-PD-1/PD-L1 and anti-CTLA-4 antibodies, and they have demonstrated promising efficacy in the treatment of CRC patients with MSI-H/dMMR (89). The KEYNOTE-177 trial enrolled 307 patients with metastatic MSI-H/dMMR CRC. Compared to chemotherapy, treatment with the anti-PD-1 antibody pembrolizumab significantly improved median PFS (16.5 vs. 8.2 months). Pembrolizumab also extended the median OS (13.7 vs. 10.8 months) (90). This outcome led to the FDA’s approval of pembrolizumab monotherapy as a first-line treatment for metastatic CRC. Furthermore, dual checkpoint blockade is also under investigation in clinical trials for MSI-H/dMMR CRC patients. The CheckMate 142 evaluated the combination of anti-PD-1 antibody nivolumab and anti-CTLA-4 antibody ipilimumab as a first-line treatment for metastatic MSI-H/dMMR CRC patients. The results reported an objective response rate of 69%, a disease control rate of 84%, and a complete response rate of 13% (91). However, only approximately 15% of patients harbor MSI-H/dMMR tumors (92). Most patients present with microsatellite stable (MSS) and mismatch repair-proficient (pMMR) tumors, a status linked to a poor response to ICIs.

Primary resistance to ICIs in MSS/pMMR CRLM arises from the synergistic action of four liver−specific barriers (1). Hepatic innate immune tolerance. LSECs and KCs constitutively express PD−L1 and secrete IL−10, TGF−β, and IDO, creating a baseline immunosuppressive state that ICIs cannot easily overcome (2, 5). Qualitative T−cell defects. Low mutational burden and scarce neoantigens lead to few tumor−infiltrating lymphocytes; residual T cells are deeply exhausted (high PD−1, TIM−3, LAG3) with low TCR affinity and impaired effector function (3, 93). Myeloid−dominated suppression. M2−like TAMs, Tregs, and MDSCs form a self−reinforcing network. PD−1/PD−L1 blockade fails to reverse MDSC−mediated arginase/iNOS activity or Treg−derived IL−10 (4, 34). Physical and mechano−immune barrier. CAF−driven extracellular matrix stiffening not only physically excludes T cells but also upregulates tumor PD−L1 via integrin−FAK−YAP/TAZ signaling; matrix stiffness directly suppresses T−cell activation through YAP (16).

Consequently, PD−1/CTLA−4 dual blockade yields objective response rates below 10% in MSS CRLM. Combination strategies targeting multiple barriers simultaneously are required to overcome resistance.

#### 3.4.2 Cell therapy and emerging immunization strategies

To overcome drug resistance, various strategies aimed at reversing the immunosuppressive niche of the liver are currently under investigation in preclinical or early-stage clinical trials. Most of these approaches are in Phase I/II, and while monotherapies have demonstrated limited efficacy, they present opportunities for combination therapy.

Cell Therapy: Early studies indicate that CEA-CAR-T therapy can achieve partial remission and induce localized necrotic responses in CRLM patients, though it carries concurrent risks of hepatotoxicity (94). Another phase I clinical trial investigating CAR-T therapy in patients with CEA-positive metastatic CRC observed certain therapeutic effects in some patients (95). However, the efficiency is limited.

LAG3 Blockade: In pMMR CRLM, TILs exhibit high LAG3 expression, and co-expression of LAG3 with PD-1 is frequently observed (96). Blockade of LAG3 can restore T cell effector functions, and clinical trials combining anti-PD-1 therapy are currently under evaluation (NCT03470922, CheckMate 142). However, LAG3 blockade alone is insufficient to overcome the multiple immunological barriers of the liver and therefore requires combination with other therapeutic strategies.

Oncolytic Viruses: NV1020 is a genetically engineered herpes simplex virus. NCT00149396 evaluated the safety and anti-tumor immune response of repeated hepatic arterial infusions of NV1020 administered before second-line chemotherapy in CRLM patients. The optimal dose was determined to be 1*10^8 pfu, at which the median OS was 11.6 months. The efficacy of NV1020 requires further validation in larger-scale phase III clinical trials, alongside exploration of its combination with chemotherapy, immunotherapy, and other modalities.

Vaccine Therapy: The phase II trial of PANVAC−V/F (CEA−MUC−1−TRICOM) combined with GM−CSF or dendritic cells (NCT00103142) evaluated its efficacy as adjuvant therapy in patients who had undergone complete resection of CRLM. The results showed 2−year recurrence−free survival rates of 81% and 50% in the DC/PANVAC and PANVAC/GM−CSF arms, respectively. However, the study had a small sample size (74 patients) and has not been validated in large−scale randomized trials. Moreover, in immunosuppressive microenvironments such as the liver, vaccine−induced immunogenicity is often attenuated, potentially necessitating combination with immune checkpoint inhibitors or metabolic modulators.

Immunotherapy has established its standard position in MSI−H/dMMR CRLM. However, for the vast majority of MSS/pMMR cases, the liver−specific immunosuppressive niche imposes multilayered barriers to therapy. Currently, most emerging strategies aimed at reversing this niche remain at early clinical or preclinical stages, and no monotherapy has yet demonstrated significant efficacy in large−scale MSS populations. Therefore, future breakthroughs are more likely to arise from rationally designed combination regimens grounded in mechanistic understanding. Various immunotherapeutic strategies are currently under investigation in clinical trials (**Table 3**).

**Table 3:** Representative clinical trials of immunotherapy for CRLM.

Trial ID	Phase	Population	Intervention	Result
NCT04166383	II	CRLM (MSS)	VB-111 + nivolumab	mPFS: 1.8 m; mOS: 6.9 m
NCT03844750	II	CRLM	Pembrolizumab + vactosertib	R0 rate:83%
NCT00149396	I/II	CRLM	NV1020 hepatic artery infusion	mOS:12.4 m (I); 11.6 m (II)
NCT00103142	II	CRLM	PANVAC-V + PANVAC-F + DC vs. PANVAC-V + PANVAC-F + GM-CSF	2-year RFS:81% vs. 50%
NCT06630624	I/II	CRLM	MWA/IRE + intra-tumoral IP-001 (novel immuno−adjuvant)	Recruiting
NCT06120127	II	CRLM	Postoperative chemotherapy + SBRT + immunotherapy	Recruiting
NCT05538195	I	CRLM	Hepatic artery infusion of anti-CEA CAR−T cells	Ongoing
NCT03844750	II	CRLM (MSS)	Pembrolizumab + vactosertib (TGF−β inhibitor)	R0 rate 83%

PANVAC-V, vaccinia-Carcinoembryonic antigen (CEA)-mucin 1 (MUC-1)- Triad of costimulatory molecules TRICOM vaccine; PANVAC-F, fowlpox-CEA-MUC-1-TRICOM vaccine; MWA, microwave ablation; IRE, irreversible electroporation.

## 4 Conclusion

CRLM remains the primary cause of mortality in CRC patients because the liver’s immune−tolerant microenvironment is hijacked by tumor cells to build a self−amplifying immunosuppressive niche. Surgery, chemotherapy, and local therapies provide short−term control, but durable responses require reversal of this niche. ICIs have succeeded in a minority of MSI−H/dMMR patients. For the MSS/pMMR cases, the liver−specific immunosuppressive niche imposes multilayered barriers that render monotherapy largely ineffective. However, emerging strategies, including cell therapy, LAG3 blockade, oncolytic viruses, and vaccines, offer hope for remodeling the hepatic immune microenvironment.

Based on an integrated understanding of the components of the immunosuppressive niche, this review proposes a dynamic three−stage model (remote priming, local conditioning, and symbiotic stabilization) that illustrates the temporal assembly of the PMN from initiation to self−reinforcement. Future therapeutic breakthroughs should be grounded in a deep understanding of the hepatic immune network, designing rational combination regimens to progressively reverse the immune tolerance within the “seed−soil” relationship. Subsequent research should employ preclinical models or single−cell/spatial transcriptomics to precisely map the complex immune interactions within this niche, identify actionable targets, and conduct well−designed translational studies to validate the clinical value of combination strategies.
